# Fast lossless compression via cascading Bloom filters

**DOI:** 10.1186/1471-2105-15-S9-S7

**Published:** 2014-09-10

**Authors:** Roye Rozov, Ron Shamir, Eran Halperin

**Affiliations:** 1 Blavatnik School of Computer Science, Tel-Aviv University, Tel Aviv, Israel; 2 Molecular Microbiology and Biotechnology Department, Tel-Aviv University, Tel Aviv, Israel; 3 International Computer Science Institute, Berkeley, CA, USA

**Keywords:** Lossless Compression, Bloom Filter, Storage, Sequencing, NGS, Alignment-free

## Abstract

**Background:**

Data from large Next Generation Sequencing (NGS) experiments present challenges both in terms of costs associated with storage and in time required for file transfer. It is sometimes possible to store only a summary relevant to particular applications, but generally it is desirable to keep all information needed to revisit experimental results in the future. Thus, the need for efficient lossless compression methods for NGS reads arises. It has been shown that NGS-specific compression schemes can improve results over generic compression methods, such as the Lempel-Ziv algorithm, Burrows-Wheeler transform, or Arithmetic Coding. When a reference genome is available, effective compression can be achieved by first aligning the reads to the reference genome, and then encoding each read using the alignment position combined with the differences in the read relative to the reference. These reference-based methods have been shown to compress better than reference-free schemes, but the alignment step they require demands several hours of CPU time on a typical dataset, whereas reference-free methods can usually compress in minutes.

**Results:**

We present a new approach that achieves highly efficient compression by using a reference genome, but completely circumvents the need for alignment, affording a great reduction in the time needed to compress. In contrast to reference-based methods that first align reads to the genome, we hash all reads into Bloom filters to encode, and decode by querying the same Bloom filters using read-length subsequences of the reference genome. Further compression is achieved by using a cascade of such filters.

**Conclusions:**

Our method, called BARCODE, runs an order of magnitude faster than reference-based methods, while compressing an order of magnitude better than reference-free methods, over a broad range of sequencing coverage. In high coverage (50-100 fold), compared to the best tested compressors, BARCODE saves 80-90% of the running time while only increasing space slightly.

## Background

Deep sequencing has become almost ubiquitous in biology over the last decade. In the past five years, sequencing costs were halved every 5 months, while storage costs were halved every 14 months [1]. The long term effect of this trend is a growing gap between our capacity to store and analyze sequencing data, and our capacity to generate such data. For sharing results of large-scale experiments, the effects have already become readily apparent: physical hard disk transfer has become a common practice, and cloud analysis platforms have been embraced in order to avoid the prohibitive time requirements needed to download or store huge volumes.

As a result, much effort has been placed on representing sequencing data more compactly. Specialized compression tools tailored to this context have emerged, improving upon general purpose compressors, such as gzip. These tools fall into two categories - reference-based [2,3], and reference-free [4-6]. The former methods utilize knowledge of the genome from which reads were extracted (with mutations and errors), while the latter use no prior information. A recent article described the Pistoa Sequence Squeeze competition, wherein the relative merits of many of these methods were compared. This article also introduced new high performance methods that were among the competition leaders [1].

Compression algorithms are evaluated by two main criteria: their compression ratio, namely, the ratios of compressed file sizes to original file sizes, and by their speed. In the context of compressing reads, compression ratios are often expressed in terms of the average number of bits per base for a fixed read length. Currently, reference-based methods generally compress most effectively, but require long run times. In order to compress reads, reference-based methods first call on a short-read aligner to find a best alignment position for each read. Such an alignment typically has only a few (or no) mismatches relative to the reference. Reads can then be represented as integers marking reference positions instead of as sequences, along with the set of differences relative to the reference. Further refinements can then be applied, such as sorting the reads by reference position and then encoding differences between consecutive positions to use fewer bits, and employing Huffman coding to encode more common mutations with less bits than rare ones [3,2]. Reference- free methods employ a variety of techniques, including boosting schemes for general purpose compressors [4,6], rough assembly for the sake of emulating reference-based compression [5], and arithmetic coding/context modeling approaches, which trade increases in run time for better compression ratios [1].

There is therefore an inherent tradeoff between runtime and compression ratio. Specifically, even though compression ratios are impressive for reference-based methods, their running times are often prohibitively high. In this work we propose a new method, Bloom filter Alignment-free Reference-based COmpression and DEcompression (BARCODE, abbreviated to BRC below), which achieves high compression ratios with a dramatic decrease of runtime. BARCODE does so by leveraging the space efficiency of Bloom filters, probabilistic data structures allowing queries of set membership. Their use has recently grown in popularity in bioinformatics [7,5,9], mainly to avoid the memory overhead needed to store large collections of k-length substrings of sequenced reads (k-mers) used to represent nodes of de Bruijn graphs in de novo assembly. To the best of our knowledge, this is the first use of Bloom filters for NGS compression.

Here, we adopt a similar scheme to that used for assembly in two recent works [8,10]. We hash whole reads into BFs as a means of compression. In tests performed, BARCODE's run times are closest to those reference-free methods while its compression ratios near those of reference-based methods. In as little as a ninth of the running time, we are able to compress to within less than 20% of the compression ratios observed for reference-based methods. We demonstrate that with higher coverage levels, BARCODE's efficiency improves, whereas reference-based methods show no improvement, while the gap in run time grows more severe. By comparing our method with several existing tools, we show its superior balance of speed and compression efficiency.

## Methods

### Technical background

A Bloom filter (BF) is an array *A *of size *m *having all positions initially marked 0. Elements are inserted into *A *by applying a collection of *h *hash functions: the output of each specifies a position to be marked with a 1 in *A*. Querying whether or not an element has been inserted involves applying the same *h *hash functions and checking the values at the positions they return. If at least one hash function returns 0, the element definitely was not inserted; if all 1s return, either it has been inserted, or it is a false positive. For a BF of size *m, n *entries can be inserted by *h *hash functions to achieve a false positive rate *F ≈ *(1 *− e*^(*−hn/m*)^)*^h^*. In [11], it is shown that for fixed *m *and *n, F *is minimized with *h = ln*(2)*ρ*, where *ρ = m/n*. Plugging this value back in for *F *leads to *F = c^ρ^*, where *c *= 0.6185.

### Encoding and decoding using a Bloom filter

Our method involves two basic processes: BF loading and querying. We initially assume all reads are unique and later relax this assumption. We load all reads into a BF *B*, and then use the reference genome to query it. We query *B *with read length subsequences (and their reverse complements) from all possible start positions on the genome. This allows us to identify all of the potential reads that correspond to genome positions, a set that covers most of the hashed reads. Some of the accepted reads will be false positives. In order to avoid them in the decoding process, we identify a set *FP *corresponding to all reads accepted by *B *that are not in the original read set. Additionally, since the reads are taken from a specimen whose genome contains mutations compared to the reference (and since sequencing is error-prone), some reads will not be recovered by querying the genome. We call this set of reads *FN. FN *and *FP *are stored separately from *B*, and compressed using an off-the-shelf compression tool. For a set of unique reads, this suffices to allow a complete reconstruction of the reads.

**Figure 1:**
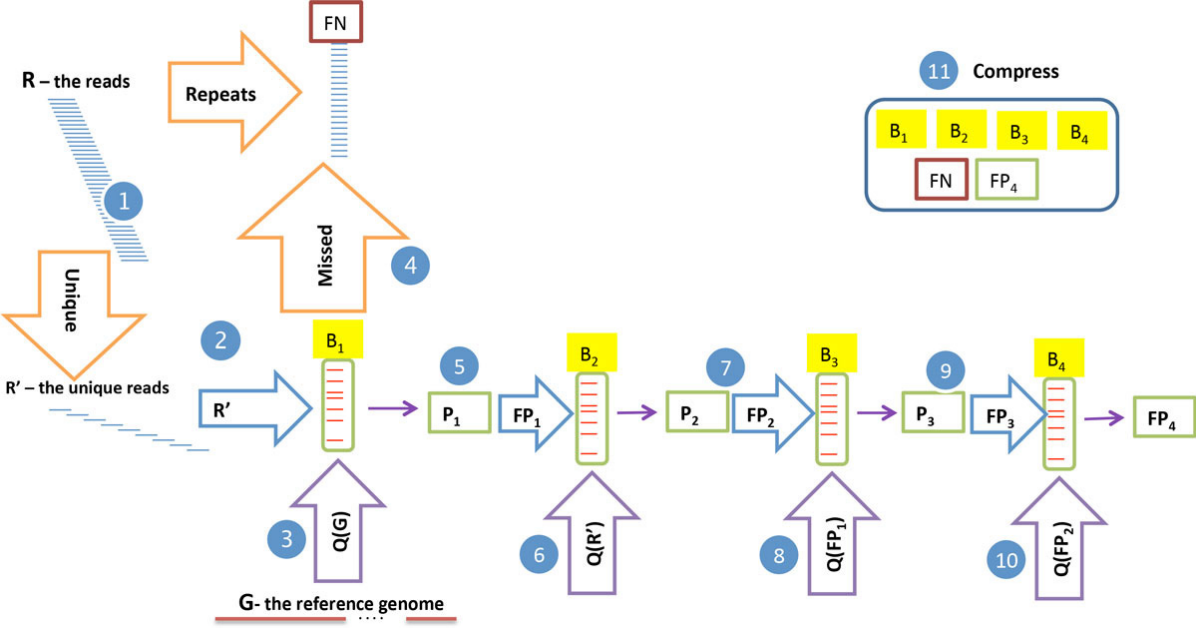
**The encoding process**. Step 1 separates the unique reads set *R' *from the repeated reads set *FN*. In step 2 unique reads (*R'*) are hashed into a BF *B*1 and the rest assigned to a set *FN*. In steps 3-4 all read-length sequences of the reference genome *G *are queried and reads accepted by *B*_1 _that are not in *R' *are added to *FP*_1_. Steps 5-10 show subsequent encoding via a BF cascade. False positives relative to each BF are input to the next BF. Each BF is then queried by using the set loaded into the last BF in the cascade. In step 11 additional compression is perfomed on the resulting BFs and sets. Orange arrows indicate assignments. Purple arrows marked with *Q*(.) indicate BF queries with sets denoted in parenthesis. Blue arrows indicate BF loading.

Decoding proceeds by decompressing *B, FN*, and *FP*, and then repeating the querying procedure. We initialize the read set to *FN*. Then, we query *B *with each position from the genome as done to identify elements of *FP*. Whenever *B *accepts we check if the accepted read is not also in *FP*, and add it to the read set if it isn't. To remove the unique read restriction, we first move all repeated reads to *FN *before loading *B*. We treat reads containing 'N' characters similarly. These two additions allow us to circumvent an inherent limitation of Bloom filters - the loss of multiplicity information - and reduces the entropy in the (now multi-) set *FN*, making it more compressible. The encoding process with one BF is detailed in steps 1-4 of Figure 1 and Algorithm 1. The relative contributions of error reads and repeated reads to *FN *are discussed in the appendix.

**Algorithm 1 **Encode one **Input**: R, G; **Output**: B, FN, FP **Conventions**: Let *g *be the length of the reference genome *G, q_i _*be the *i^th ^*genome query, *ℓ_read _*be the sequenced read length, and *P *be the set of genome queries accepted by *B*. For brevity, we exhibit queries from only the forward strand, whereas our implementation queries (and accepts from) both strands.

FN := {*r *: *r ∈ R *and (*r *is repeated in *R *or *r *contains an 'N')}

R' := R \ FN

for all *r ∈ R' *do

insert(*r, B*)

end for

**for all ***i ∈ *[1, *g − ℓ_read _*+ 1] **do**

**;if ***qi ∈ B ***then**

P := P ∪ q_i_

end if

end for

FN := FN ∪ {R' \ P}

FP := P \ R'

return (*B, FN, FP *)

### Encoding and decoding using a BF cascade

Although appealingly simple, we found the above method did not offer competitive compression, as the costs imposed encoding *FP *and *FN *outweighed the benefit of storing the unique reads in *B*. Thus, to reduce the number of false positives that need to be compressed separately, we use a cascade of BFs as in [10]. To this end, we rename *B *and *FP *above as *B*_1 _and *FP*_1_, respectively. We consider *B*_1 _to be the first BF in a cascade, and each element of *FP*_1 _is then hashed into a subsequent BF *B*_2_. We note that since *B*_2 _is meant to store false positive reads it should reject true reads: thus, any element of *R' *(the set of unique reads) accepted by *B*_2 _is a false positive relative to *B*_2_. Thus, to identify *FP*_2_, we add each element accepted by querying *R' *against *B*_2_. This process can be continued for any desired number of BFs. Once BFs are loaded in this way, to identify real reads, we query each BF in the cascade and accept reads only if the index of the first BF to reject them is even.

Since elements inserted to *BF_j _*are necessarily a subset of those inserted to *BF*_*j−*2_, we see an exponential drop-off in BF size (since F is fixed). Since sizes for successive BFs alternately depend on *n *and (2*g − n*)*ρF ≈ *2*gρF *(the number of false positives expected for 2*g *queries from *G *multiplied by the cost per element, assuming *g >> n*), we expect the total file size to be approximately (*nρ *+ 2*gρF *+ *nρF *+ 2*gρF*^2 ^+ *..*.) bits. Using *F = c^ρ ^*from above, we observe that for an infinite cascade, the average number of bits per read is then

(1) $$\left(\rho \frac{2g\rho c^{\rho }}{n}\right)\left(1+c^{\rho }+c^{2\rho }+...\right)=\left(1+\frac{2gc^{\rho }}{n}\right)\left(\frac{\rho }{1-c^{\rho }}\right).$$

**Figure 2:**
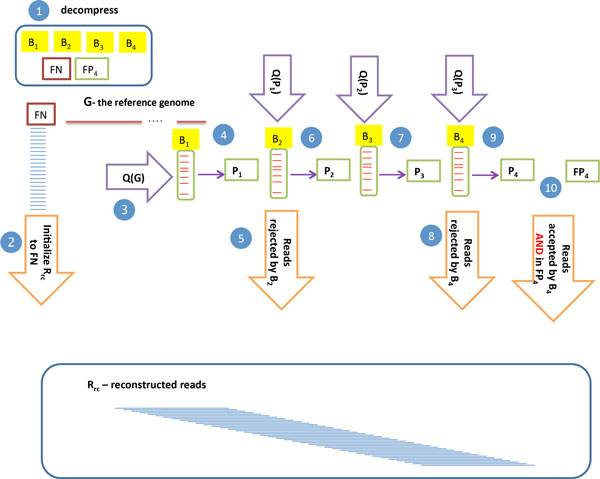
**Decoding the reads**. Following decompression of BFs, *FP*_4_, and *FN*, BF querying commences. Each read accepted by a BF is used to query subsequent BFs until rejection. Reads rejected by even BFs or accepted by *B*_4 _and in *FP*_4 _are added to the reconstructed reads, *R_rc_*. Purple arrows are consistent with Figure 1. Orange arrows indicate additions to *R_rc_*, the reconstructed reads.

Here the left hand side represents the sum of costs due to the expected number of elements in each BF for an infinite cascade. In practice, we use four BFs and a numerical solver in scipy [12] employing the L-BFGS-B [13] algorithm to find the value of *ρ *minimizing the above expression. The small list *FP*_4 _is encoded separately along with *FN*. The process is described in Figure 1 steps 5-11 and Algorithm 2. Decoding proceeds using queries from *G *as before, but in this case each accepted read is used to query subsequent BFs until rejection. This is depicted in Figure 2and Algorithm 3.

**Algorithm 2 **Encoding Let *B_j _*be the *j^th ^*BF loaded (*j ∈ *[2, 4]) with *FP*_*j*-1_, *S *∩ B*_j _*is short-hand notation for the subset of *S *accepted by *B_j_*.

   (*B*_1_, *FN, FP*_1_) := **Encode one**(*R, G*) # we initialize by calling Algorithm 1

   F P_2 _:= R' ∩ B2

   **for ***j *= 3 **to ***j *= 4 **do**

      **for all ***r ∈ FP*_*j−*1 _**do**

         insert(*r, B_j_*)

      **end for**

      FP_j _:= FP_j−2 _∩ B_j_

   **end for**

   return (*B*_1_, *B*_2_, *B*_3_, *B*_4_, *FN, FP*_4_)

**Algorithm 3 **Decoding **Input**: (*B*_1_, *B*_2_, *B*_3_, *B*_4_, *FN, FP*_4_, *G*); **Output**: (*R_rc_*) For brevity, reconstruction of only one strand is shown.

   R_rc _:= FN

   **for all ***i ∈ *[1, *g − ℓ_read _*+ 1] **do**

    **for ***j *= 1 **to ***j *= 4 **do**

      **if **$q_{i}\mathrm{\varepsilon ̸}B_{j}$**then**

        **if **j is even **then**

          R_rc _:= R_rc _∪ q_i_

        **end if**

      continue {increment i}

    **end if**

   **end for**

   **if **j = 4 **and ***q_i _∈ FP*_4 _**then**

    R_rc _:= R_rc _∪ q_i_

   **end if**

  **end for**

  return *R_rc_*

### Additional compression

BF parameters are automatically set to make each BF more compressible. This involves incrementing the number of hash functions for each BF from 1 to the minimal number that allows it to both have an uncompressed file size lower than a preset threshold (we used 500 MB) and obtain the value of F from equation 1. Typically, this results in h being in the range of 1 to 3. We do this in order to reduce each BF's compressed size (at the expense of increasing its RAM occupation); this practice is introduced in [11].

Once BFs are loaded and the sets *FP*4 and *FN *are identified, we use 7zip [14] to compress the *B*1, ..., *B*4 and SCALCE [4] to compress the output lists *FP*_4 _and *FN*. In principle, any general compression tool can be used for the BFs, and it is preferable to use a tool that takes advantage of existing sequence overlaps among the leftover reads to compress them efficiently.

## Results and discussion

### Comparison on simulated reads

We simulated reads from Human (hg19) chromosome 20 using dwgsim [15]. This tool introduces mutations into the reference genome and then samples reads from both genome strands using a user-defined per base error rate. We sampled 100 bp single end reads at various coverage levels with a 0.001 mutation rate and a per base error rate increasing from 0 to 0.005 from the 5' to the 3' end of reads (in line with current estimates of Illumina error rates [16]). We also demonstrated the effect of varying the error rate In Figure 4. All reported results were run on a 16 core AMD Opteron 6140 (2.6 GHz) 128 GB RAM server, running the Ubuntu 12.04 Linux operating system.

**Figure 3:**
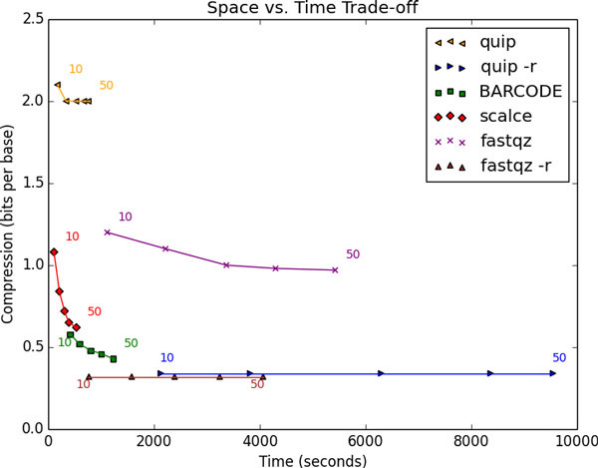
**A comparison of sequence compressors**. The figure shows elapsed real run time vs. compression ratios of read sequences in bits per base for read length 100 bp. The measurements of each method for different coverage levels are connected by a line. Points correspond to coverage levels from 10 to 50 in multiples of 10 from left to right. Methods denoted with an "-r" were run with the reference-based option. Run times were measured with /usr/bin/time using a single thread on the same Linux server.

**Figure 4:**
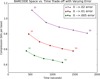
**The effect of varying error rate**. BARCODE runs are shown with error rates varying from 5' to 3' ends as indicated in the figure legend. Compression ratios increase with greater error, but higher coverage compensates somewhat. Overall, as coverage increases, run time is effected by increasing error more than compression ratios, as can be seen by the decreasing slopes between fixed coverage points between the green and red curve and between the red and purple curves.

We found that BARCODE compresses more effectively at higher coverage. Although the proportion of reads in *FN *increases as the proportion of unique reads decreases (Table 1), BARCODE benefits from SCALCE's increasing efficiency due to greater redundancy among FNs. BARCODE's decode times were similar to its encode times, as would be expected since both rely on the same genome querying procedure.

**Table 1:** BRC performance with varying coverage.

coverage	time (sec)	*|R| *(M)	*|FP*4*| *(K)	*|FN | *(M)	BF size (MB)	*FP*_4 _size (MB)	*FN *size (MB)	compression bits/base
10	410	6.3	3.7	2.0	8.5	0.38	36.8	0.58
20	590	12.6	6.8	4.4	14.2	0.68	66.8	0.52
30	800	18.9	9.3	7.1	19.0	0.94	93.8	0.48
40	1006	25.2	20	10.2	22.8	2.01	119.0	0.46
50	1220	31.5	16	13.5	26.4	1.66	143.0	0.43

Reads were simulated from hg19 chromosome 20 with 100 bp single end reads. A mutation rate of 0.001 was used along with 0-0.005 per base error rate along the length of each read. Run times include additional compression steps performed by SCALCE and 7zip in single thread mode. *R *- the read set. *FP*_4 _- the final false positive set. *FN *- the set of reads not encoded by the BFs.

To demonstrate that our use of BFs improves upon SCALCE's compression results, we compared our results with SCALCE run on all reads. We also tested quip [5] and fastqz [1], state-of-the-art tools in terms of both compression efficiency and speed [1]. All three tools either output compression results or ratios separately for sequences, read names, and quality scores. We note that the best performers in the Sequence Squeeze competition in terms of base compression ratio, Sam-comp and CRAM, did not provide such outputs and thus did not allow direct comparison. Quip and fastqz also include both reference-based and reference-free modes. We performed alignment via bowtie2 [17] for quip runs while fastqz performed its own alignment. To ensure a fair comparison, all tools were run as a single thread when possible, including calls to 7zip and SCALCE from BARCODE. Fastqz used three threads during its run, as this was not a user-selectable parameter, but each thread was assigned to one of sequences, qualities, and names. Figure 3 compares BARCODE with other tools in terms of run time and compression efficiency. A full listing of program parameters used is provided in Table 2.

**Table 2:** Program parameters used in compression tool comparison (Figure 3)

Program	Parameters
dwgsim	-C coverage level -H -e 0.0-0.005 -R 0.0 -1 read length -2 0 -y 0.0
bowtie2	-x chr20 -U input fastq -S
SCALCE	input fastq -T 1 -A -n library -o output prefix
quip (default)	-o=quip -i=sam input sam
quip (reference)	-o=quip -r ref fa -i=sam input sam
fastqz (default)	c input fastq output prefix
fastqz (reference)	c input fastq output prefix r packed ref file
BRC	rec load bf -err rate 0 -e 0 -i 4 reads file packed ref file

Overall, we found the compression ratio improved with greater coverage for all reference-free methods, and remained essentially constant for reference-based methods. Figure 3 shows that reference-based compressors are better in compression ratios but reference-free compressors are faster (An exception to this trend was fastqz, whose reference-based version is faster than its reference-free version, likely due to the use of context model-based arithmetic coding). Quip performed poorly in compressing sequences without a reference, showing it has apparently been optimized for speed and perhaps compression of qualities and read names. SCALCE shows strong dependence of compression ratio on coverage, as would be expected by its leverage of the recurrence of long subsequences. BARCODE takes advantage of this trend to also improve with higher coverage, even as the proportion of reads hashed to BFs decreases (See Table 1). BARCODE's times are closest to SCALCE and reference-free quip, and its compression ratios approach those of reference based methods, especially at higher coverage values. For most coverage values, it maintains an order of magnitude time advantage vs. reference-based methods (~2-3x vs. fastqz, ~5-7x vs. quip), as well as an order of magnitude compression advantage of the tested reference-free methods.

### Higher coverage, longer reads

We tested scenarios of higher coverage and longer read lengths: (1) coverage 100 and read length 100 bp, (2) coverage 100 and read length 200 bp, and (3) coverage 200 and read length 400 bp. Table 3 shows a continuation of the trends expressed at lower coverage levels. Higher coverage aided reference-free methods, but not reference-based methods. Longer reads improved compression ratios in each case with the exception of fastqz -r. We observed larger impacts on run time as a result of doubling read length than coverage.

**Table 3:** Performance comparison on high coverage values and read lengths longer than 100.

Time (sec)	coverage	quip	scalce	fastqz	BRC	quip -r	fastqz -r
	50	759	533	5426	1220	9544	4063
	100	1599	1016	10417	2211	20216	7479
	100, len 200	1280	1165	8760	1400	21705	7400
	200, len 400	2284	2518	15706	2209	51628	17769
Compression (bits/base)	50	2.0	0.62	0.97	0.43	0.34	0.32
	100	2.0	0.53	0.58	0.40	0.34	0.32
	100, len 200	1.8	0.43	0.61	0.37	0.19	0.45
	200, len 400	1.7	0.32	0.41	0.31	0.12	0.41

Reads were generated as described in the main text. Quip -r times include bt2 alignment.

## Conclusions

We have presented a new approach to compressing sequencing reads, bridging the gap between the speed of alignment-free, reference-free methods and the compression efficiency of reference-based methods. We have tested the dependence of extant sequence compressors on coverage levels and shown that while reference-based methods compress most efficiently, they place a heavy burden on CPU times due to alignment and cannot leverage added redundancy to benefit compression ratios. Reference-free methods do benefit from higher coverage, but maintain a considerable distance from reference-based methods in terms of compression ratios even at the highest levels tested. Although we have shown that our new method, BARCODE, obtains a better trade-off than either of these extremes, we maintain that there remains much room for improvement, even when considering the inherent constraints imposed by the Kolmogorov complexity of the data. We note that further comparison to other methods like CRAM [3] and sam_comp [1] is needed.

BARCODE is currently a proof-of-principle implementation, and thus we expect that further optimization will improve run time and compression efficiency. Compression ratios may be improved by taking advantage of better general compression tools available such as the ZPAQ library [18], as fastqz and sam_comp do. Thus far, we have not utilized arithmetic coding techniques because they employ multiple threads and thus introduce significant additional resource requirements. Our approach can also be extended to allow for fast access to variants in the original data by using conventional BFs that are not compressed, and by compressing FN/FP reads using encoding that allows fast random access (at some expense of compression ratio). We aim to investigate these paths in the future.

## Appendix

### Real data test

We examined BARCODE's performance on the C. Elegans data set tested in the Sequence Squeeze competition, SRR065390_1. This data set consists of 33415360 100 bp reads, amounting to 33-fold coverage of the genome. BARCODE's compression ratio on this data was 0.46 bits per base, and run time was 1203 seconds, in line with experiments described in the main text and comparable with reference-based methods tested in the Sequence Squeeze competition [1].

### Contributions of repeated reads vs. errors to FN

FN is comprised of repeated reads filtered out to preserve their multiplicities, and reads differing from the reference because of errors or variations. Here, we describe the relative contributions of each part. The expected number of repeated reads can be described probabilistically. Assuming reads are sampled independently from *G*, given a read *r*, the probability of drawing *r *again is $1-\frac{1}{G}$. For *R *reads, the probability of observing no repetitions is then ${\left(1-\frac{1}{G}\right)}^{R-1}$. Thus, the expected number of repeated reads is $R\left(1-{\left(1-\frac{1}{G}\right)}^{R-1}\right)$. Since we hash reverse complement reads from reference strands separately, we revise the length considered to 2*G*. Since we wish to count the total multiplicity of each repeated read, the contribution of repeated reads to *FN *is thus approximated by $2R\left(1-{\left(1-\frac{1}{2G}\right)}^{R-1}\right)$. Clearly, this contribution depends on coverage, as shown in Table 4.

**Table 4:** Counts of repeats vs. errors with increasing coverage. The proportion of reads due to errors remains roughly constant, while the proportion due to repeats increases as coverage increases.

Coverage	*|R| *(M)	repeats (M)	*|FN| *(M)
10	6.3	0.3	2.0
20	12.6	1.3	4.4
30	18.9	2.8	7.1
40	25.2	4.9	10.2
50	31.5	7.4	13.5

errors with increasing coverage. The proportion of reads due to errors remains roughly constant, while the proportion due to repeats increases as coverage increases.

We model the contribution of error to *FN *using *Binom*(100, *p*) with *p *= 0.0025, the mean error over the read length used in our simulated reads (where error varies from 0 to 0.005 from the 5' to 3' ends). Most of the mass is carried by the one and two error terms, leading to a relative error proportion estimate of $\left(\begin{array}{c} 100\\ 2 \end{array}\right){\left(1-p\right)}^{98}p^{2}+\left(\begin{array}{c} 100\\ 2 \end{array}\right){\left(1-p\right)}^{99}p$. Table 4 shows this proportion is independent of coverage level.

## Availability

BARCODE can be downloaded at http://www.cs.tau.ac.il/~heran/cozygene/software.shtml

## List of abbreviations

BF- Bloom filter; BRC - BARCODE; bpb - bits per base; bp - base pairs

## Competing interests

The authors declare that they have no competing interests.

## Declarations

RS was supported in part by the Israel Science Foundation (grant 317/13) and by the Raymond and Beverly Sackler chair in bioinformatics. RR was supported in part by a fellowship from the Edmond J. Safra Center for Bioinformatics at Tel-Aviv university, and by the Center for Absorption in Science, the Ministry of Immigrant Absorption in Israel. EH is a faculty fellow of the Edmond J. Safra Center for Bioinformatics at Tel Aviv University. EH was partially supported by the Israeli Science Foundation (grant 1425/13), and by National Science Foundation grant III-1217615.

This article has been published as part of BMC Bioinformatics Volume 15 Supplement 9, 2014: Proceedings of the Fourth Annual RECOMB Satellite Workshop on Massively Parallel Sequencing (RECOMB-Seq 2014). The full contents of the supplement are available online at http://www.biomedcentral.com/bmcbioinformatics/supplements/15/S9.

## Authors' contributions

RR developed the method. RS and EH designed the experiments. RR implemented the method and performed experiments. All authors analyzed results, co-wrote the manuscript, and read and approved the final manuscript.
